# Cerebrovascular Events in Suspected Sepsis: Retrospective Prevalence Study in Critically Ill Patients Undergoing Full-Body Computed Tomography

**DOI:** 10.3389/fneur.2022.811022

**Published:** 2022-05-09

**Authors:** Julian Pohlan, Jawed Nawabi, Denis Witham, Luna Schroth, Finn Krause, Jan Schulze, Simon Gelen, Robert Ahlborn, Kerstin Rubarth, Marc Dewey

**Affiliations:** ^1^Department of Radiology, Charité – Universitätsmedizin Berlin, Corporate Member of Freie Universität Berlin, Berlin Institute of Health, Charité Universitätsmedizin Berlin, Humboldt-Universität zu Berlin, Berlin, Germany; ^2^Berlin Institute of Health, Charité Medical University of Berlin, Berlin, Germany; ^3^Department of Cardiology With Intensive Care, Charité – Universitätsmedizin Berlin, Charité Universitätsmedizin Berlin, Berlin, Germany; ^4^Department of Information Technology, Charité – Universitätsmedizin Berlin, Charité Universitätsmedizin Berlin, Berlin, Germany; ^5^Institute of Biometry and Clinical Epidemiology, Charité – Universitätsmedizin Berlin, Corporate Member of Freie Universität Berlin, Charité Universitätsmedizin Berlin, Humboldt-Universität zu Berlin, Berlin, Germany

**Keywords:** sepsis, computed tomography, cCT, cerebrovascular events, stroke

## Abstract

**Purpose:**

This study aimed at retrospectively evaluating full-body computed tomography (CT) examinations for the prevalence of cerebrovascular events in patients with suspected sepsis treated in the intensive care unit (ICU).

**Methods:**

All full-body CT examinations, i.e., both cranial CT (cCT) and body CT including chest, abdomen and pelvis, for focus search in septic patients over a 12-months period were identified from three ICUs, using full-text search. From this retrospective cohort, we fully analyzed 278 cCT examinations for the occurrence of acute cerebral findings. All acute cerebrovascular events were independently reviewed by two blinded readers. Clinical and laboratory findings were extracted. The data were statistically analyzed using contingency tests.

**Results:**

In our population of patients with suspected sepsis, 10.8% (*n* = 30/278) were identified to have major cerebral events, including 7.2% (*n* = 20/278) major cerebrovascular events and 4.3% (*n* = 12/278) generalized parenchymal damage. 13.4% (*n* = 22/163) of patients with a severe coma as compared with non-severe coma, 4.4% (*n* = 3/68), showed a major cerebral event (*p* = 0.04). Patients referred from the cardiology/nephrology ICU ward showed major cerebral events in 16.3% (*n* = 22/135), as compared with 4.9% (*n* = 3/61) in patients from pulmonary ICU and 6.1% (*n* = 5/82) major cerebral events with surgical referral (*p* = 0.02).

**Conclusion:**

Our study provides further evidence that septic patients may suffer from cerebral events with relevance to their prognosis. Severe coma and the referring ward were associated with acute cerebral conditions. Full-body CT has the advantage of both detecting of septic foci and possibly identifying ischemic or hemorrhagic stroke in this vulnerable patient population.

## Introduction

Sepsis is a serious condition defined as infection-related organ dysfunction (1). The foremost aim of treatment is to rapidly eliminate infectious foci (1, 2). As sepsis-associated encephalopathy (SAE) is common and may hamper both taking an adequate patient history and performing a comprehensive physical examination, imaging plays an important role in identifying the source of infection (1, 3). The prognostic relevance of the neurological condition in sepsis has been discussed before, and modifiable risk factors for SAE have previously been identified (4). It has also been reported that sepsis may mimic stroke (5).

Coagulopathy is a common complication in sepsis and seriously affects patient outcome when culminating in disseminated intravascular coagulation (6). There is growing evidence of an association between sepsis and cerebrovascular events (7–9). Both stroke and intracranial hemorrhage continue to have a high morbidity and mortality (10, 11). Although initially established to evaluate trauma patients, the Glasgow Coma Scale (GCS) can also be used to assess the level of consciousness in patients with non-traumatic coma states (12). In comatose patients, an association between a low GCS score and sepsis has been established (13).

Whereas there are no specific imaging recommendations for patients with sepsis, computed tomography (CT) is typically used when examining patients under emergency conditions (1). Body CT has been shown to reliably identify infectious sources in septic patients (14, 15). The authors previously conducted a detailed and systematic analysis of the role of CT for focus identification in sepsis in various hospital settings (16–18). Several studies have demonstrated a high diagnostic yield of non-contrast cranial CT (cCT) for cerebral pathologies including ischemic and/or hemorrhagic stroke (19, 20).

There is growing evidence to suggest that cerebrovascular events play a role in the outcome of septic patients. We therefore conducted a study aimed at identifying cerebrovascular events in patients with suspected sepsis who undergo full-body CT.

## Methods

### Patient Selection

**Figure 1:**
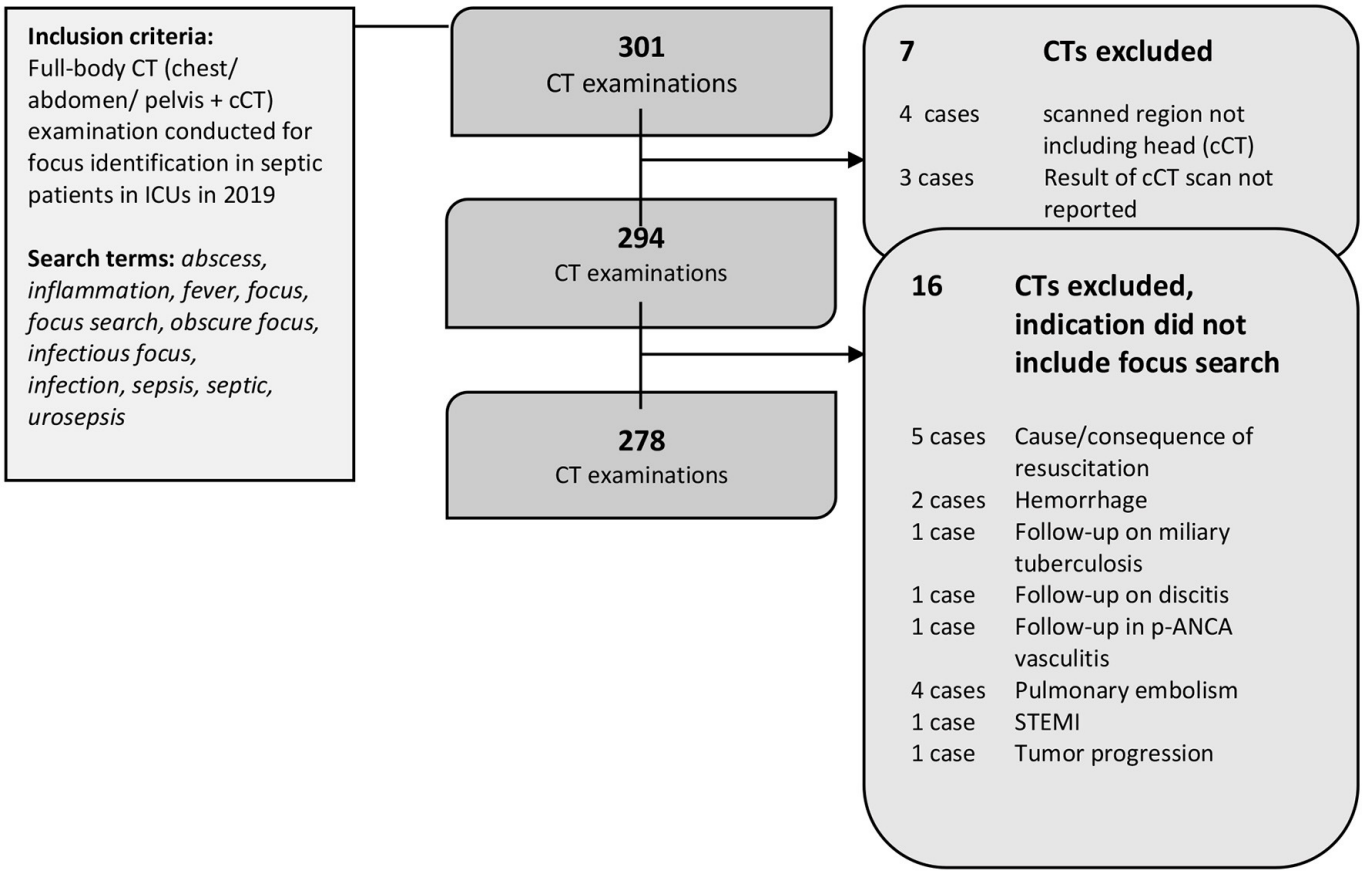
Patient flow. 301 CT reports were identified by the full-text search. Seven CTs were excluded as the required organ regions were not fully examined. Another 16 CTs were excluded after the analysis due to indications other than focus search. A final 278 CT reports were included in the analysis.

Full-body CTs performed in patients from three large ICUs between Jan. 1, 2019 and Dec. 21, 2019 were included in this retrospective cohort study (Figure 1). A full-text search for CT reports was conducted using the following key words: abscess, infection, fever, focus, infectious focus, search for focus, obscure focus, sepsis, urosepsis, and septic. Another analysis of data from 2018 with the same approach was previously published (16–18). As the query was designed to be broad, not all terms needed to be present. As reported below, patients were later controlled for assessment of organ dysfunction to account for the diagnostic criteria of a diagnosis of sepsis. All reports on CT examinations performed to search for an infectious focus in the chest, abdomen, and pelvis in adult patients aged >18 years who also underwent a cCT were considered for analysis (Figure 1). Approval from the local ethics committee was obtained (EA1/100/19). All analyses were performed respecting the Declaration of Helsinki.

### Data Collection

The CT reports were collected and analyzed in detail after the exclusion of 23 reports as explained below. In addition to the radiological findings, we automatically extracted clinically relevant data from bedside ICU software (COPRA, COPRA System GmbH, Berlin, Germany) based on the specific case numbers. First, we retrieved laboratory data such as infection and coagulation parameters: international normalized ratio (INR), prothrombin time (PT), activated partial thromboplastin time (aPTT), thrombocytes, lactate, C-reactive protein (CRP) and procalcitonin (PCT). Second, we extracted clinical scores as the Sequential Organ Failure Assessment (SOFA) and Glasgow Coma Scale (GCS). SOFA allowed to control for organ dysfunction as one major aspect of the diagnosis of sepsis in this cohort. Thirdly, the following outcome parameters were collected: in-house mortality, length of hospitalization and duration of intensive care.

### Review of CT Reports

The cCT findings were further analyzed and categorized as major or minor cerebral findings. Acute cerebrovascular findings such as hemorrhagic or ischemic stroke were categorized as major cerebrovascular findings. A comprehensive list of the categories is provided in the supplements of this manuscript (Supplementary Table 1). Image findings were further classified as major vs. minor events: acute cerebrovascular events and generalized parenchymal damage were classified as major cerebral events. In two cases, cCT showed both a cerebrovascular event as well as generalized parenchymal damage.

### Imaging

All CT examinations included were performed during clinical routine. When cerebrovascular events were suspected, non-contrast cCT was performed first. Next, an iodine-based contrast agent was administered after 60–80 seconds for focus search or in a two-phased protocol with an arterial phase (triggered to the pulmonary trunk or aorta, when additional events were discussed, e.g., pulmonary embolism) for the chest and upper abdomen, followed by a venous phase after 60–90 s for the abdomen and pelvis. CT angiography of the head and neck was performed, when focal neurological deficits or coma were presumed to be due to ischemic stroke. CT venography after 40 seconds was run when sinus thrombosis was suspected. A delayed post contrast cCT scan was performed after 300 seconds when a condition like cerebral abscess was suspected. Routinely, 1 mm and 5 mm 3-dimensional reconstructions were generated. A total number of 278 cCT scans were included in the analysis. Of these, 74.5% were non-contrast cCTs (*n* = 217/278). In 23.7% (*n* = 66/278) non-contrast scans were followed by post contrast cCT. Thirdly, in 1.8% (*n* = 5/278) non-contrast cCT was followed by CTA.

### Rating

All acute cerebrovascular events detected by non-contrast cCT were independently reviewed for ischemic/hemorrhagic stroke, type of bleeding, vascular territory, and size by two raters with 5 years of experience in stroke imaging in a blinded assessment (Supplementary Table 1). Any discrepancies were jointly resolved on the basis of the original report as well as follow-up imaging, e.g., magnetic resonance imaging (MRI) if available.

### Statistical Analysis

All data were collected in Excel (Microsoft, Office 365 MSO, Microsoft, Redmond, WA, USA, Version 1908, 2016). Data protection was guaranteed. Analysis was performed using SPSS (IBM Statistics software, IBM Corporation, Version 25, 2017). Contingency tables were used for assessment of categorical data, with chi-squared tests (i.e. Pearson's chi-squared test) used for calculations. All analyses were performed as two-sided tests. A *p* < 0.05 was considered statistically significant. Due to the exploratory characteristic of this study, no adjustment for multiplicity was performed.

## Results

### Study Population

This retrospective study fully analyzed reports of 278 CT examinations conducted in a total of 213 patients (Table 1). Of those, 38.5% (*n* = 82/213) were women. Mean patient age was 64.4 years (SD 12.8). Per patient, one (78.4%; *n* = 167/278), two (14.1%; *n* = 31/278), three (5.6%; *n* = 11/278) or four (1.9%; *n* = 4/278) full-body CT examinations, including both non-contrast cCT for detection of cerebral events and contrast-enhanced imaging of the chest, abdomen, and pelvis for infectious focus search. All referrals were from the ICU, thereof 29.5% (*n* = 82/278) from a surgical and 70.5% (*n* = 196/278) from a medical ICU. Of the latter, 21.9% (*n* = 61/278) were referred from the pulmonary/infectious diseases ICU and 48.6% (*n* = 135/278) from the cardiology/nephrology ICU. 38.5% (*n* = 82/213) of patients died during their hospitalization. In the subgroup of survivors, patients had a mean total hospitalization time of 59.3 days (SD 65.9). Patients required intensive care for a mean of 8,897 h (SD 1,657).

**Table 1:** Patient characteristics; a total of 213 patients were analyzed. Further analysis included 278 CT examinations.

**Variable**		**Total or mean number**	**Percentage or SD**
Patients		213	
Sex	Female	82 patients	38.5%
	Male	131 patients	61.5%
Age	Female	65.8 years	SD 13.5
	Male	63.6 years	SD 12.4
	Combined	64.4 years	SD 12.8
Total hospitalization period		47.7 days	SD 57.6
Total duration of intensive care		996 h	SD 1400.3
Hospitalization in survivors		59.3 days	SD 65.9
Intensive care in survivors		8,897 h	SD 1657.5
In-house mortality	Deceased	82 patients	38.5%
	Survived	131 patients	61.5%

*SD, standard deviation*.

### Imaging Findings

In this study population of septic patients, 10.8% (*n* = 30/278) of the examinations showed at least one major cerebral finding (Supplemental Figure 1): major cerebrovascular events accounted for 7.2% (*n* = 20/278) and generalized parenchymal damage for 4.32% (*n* = 12/278). In two cases the CT-examination showed both a cerebrovascular event as well as generalized parenchymal damage. Major cerebrovascular events were ischemic strokes in 20.0% of cases (*n* = 4/20) and intracranial bleedings in 80.0% (*n* = 16/20) (Table 2). Analyzing the causalities in all patients with generalized parenchymal damage, 90.0% (*n* = 9/10) of patients were found to have suffered from hypoxia related to resuscitation in the course of cardiac arrest with the remaining one patient, i.e.,10.0% (*n* = 1/10), having suffered from hypoxia related to a near drowning incident in cold water.

**Table 2:** CT examinations.

**Variable**		**Total or mean number**	**Percentage**
Cases		278	
Body CT^a^	Contrast	270	97.1%
	Non-contrast	3	1.1%
	Information missing	4	1.4%
cCT	Non-contrast (nc)	217	74.5%
	Nc-cCT plus post contrast scan	66	23.7%
	Nc-cCT plus CT angiography	5	1.8%
cCT Findings^b^	No findings	171	61.5%
	Acute cerebrovascular event	20	7.2%
	Mass lesion	0	0%
	Generalized parenchymal damage	12	4.3%
	Atrophy	11	4.0%
	White matter disease	39	14.0%
	Vasosclerosis	4	1.4%
	Small mass lesion	4	1.4%
	Ventricular pathology	7	2.5%
	Chronic vascular pathology	10	3.6%
	Other	18	6.5%
Septic Focus	Lung	198	71.2%
	Abdomen	36	12.9%
	Other	15	5.4%
	No focus detected	29	10.4%

a *Body CT includes CT of chest, abdomen, and pelvis*,

b *Multiple findings in one CT Scan possible*.

### Predictors of Imaging Findings

**Figure 2:**
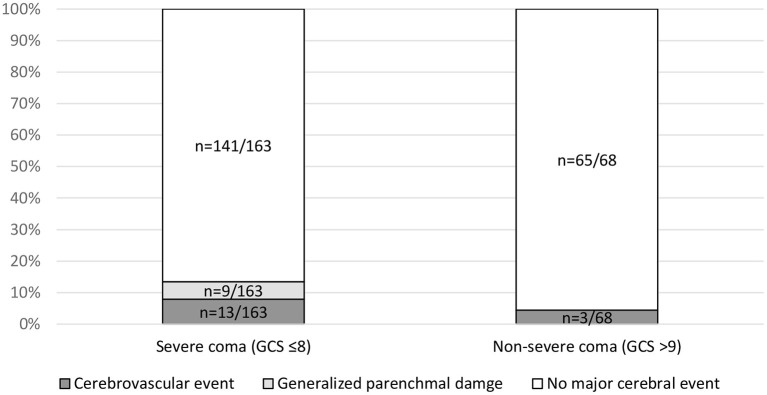
Association of major cerebral events and Glasgow Coma Scale (GCS). Subgroups with severe coma (GCS ≤8) vs. non-severe coma were compared using a contingency table analysis for the parameters cerebrovascular events (*p* = 0.30), generalized parenchymal damage (*p* = 0.05), and major events (*p* = 0.04). GCS was available in 231 cases.

The association of three clinical parameters with major cerebral imaging findings was analyzed: patients with severe coma (GCS ≤8) showed significantly more major cerebral events as compared with non-severe coma (GCS >9), i.e., 13.4% (*n* = 22/163) vs. 4.4% (*n* = 3/68) with *p* = 0.04, respectively (Figure 2). In detail, 5.5% (*n* = 9/163) of patients with severe coma (GCS ≤8) showed generalized parenchymal damage and 7.9% (*n* = 13/163) suffered a cerebrovascular event. In the subgroup of patients with non-severe coma, there were no cases of generalized parenchymal damage (*n* = 0/68; *p* = 0.05) and cerebrovascular events in 4.4% (*n* = 3/68; *p* = 0.30).

**Figure 3:**
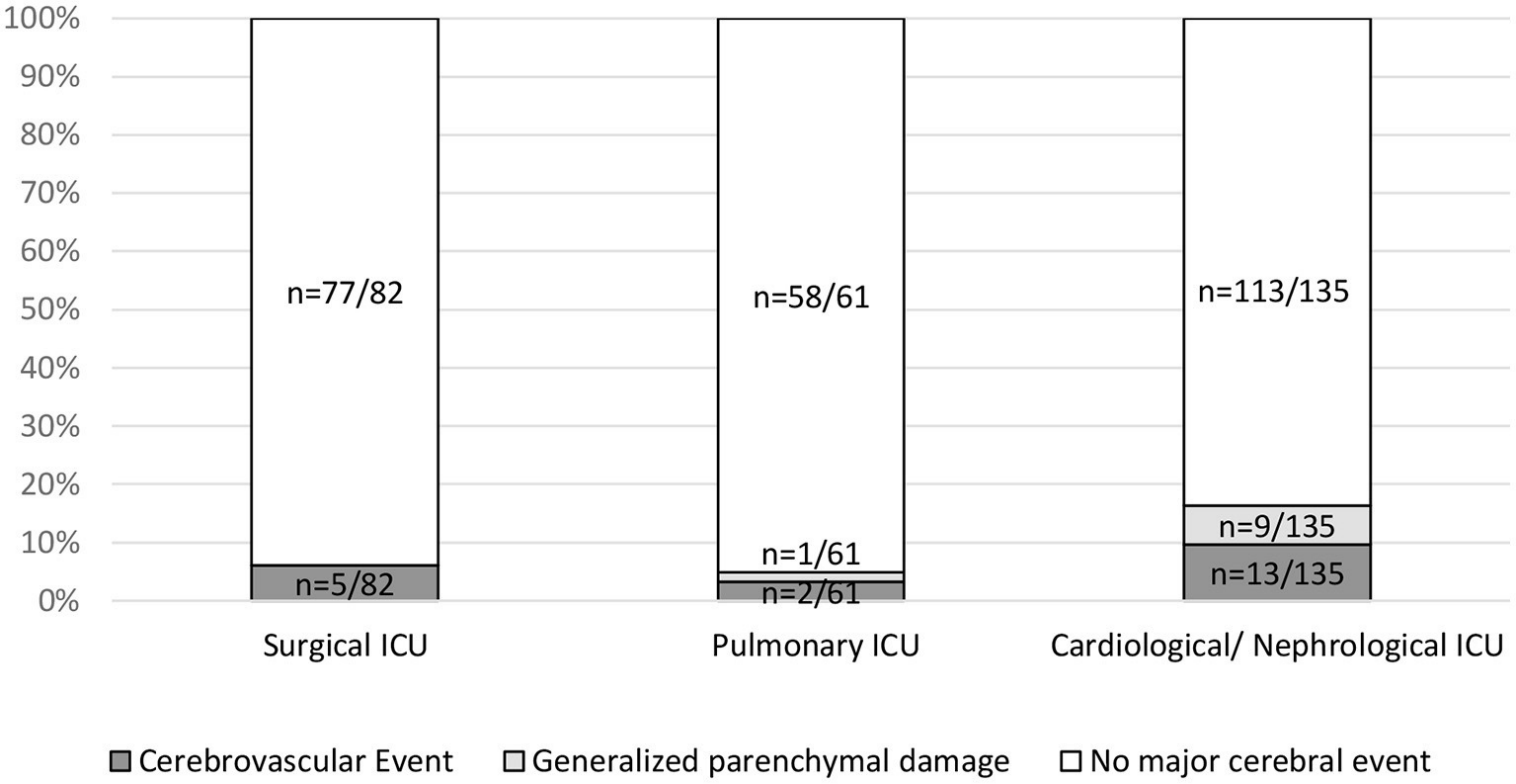
Association of major events with referring ICU. Referring ICUs (Surgical ICU, Pulmonary ICU and Cardiological/Nephrological ICU) were compared using a contingency table for the parameters cerebrovascular events (*p* = 0.25), generalized parenchymal damage (*p* = 0.03), and major events (*p* = 0.02).

As an additional clinical parameter, the referring ward (medical vs. surgical) was found to be significantly associated with major cerebral events (*p* = 0.02). Patients referred from the cardiology/nephrology ICU ward showed major cerebral events in 16.3% (*n* = 22/135), as compared with 4.9% (*n* = 3/61) in patients from the pulmonary ICU and 6.1% (*n* = 5/82) in patients from the surgical ICU. A significant association was established between generalized parenchymal damage and the referring ward (*p* = 0.03) with generalized parenchymal damage in 6.7% (*n* = 9/135) of cases referred from the cardiology/ nephrology ICU ward vs. 0% (*n* = 0/82) and 1.6% (*n* = 1/61) of cases referred from the surgical and pulmonary ward, respectively (Figure 3).

**Figure 4:**
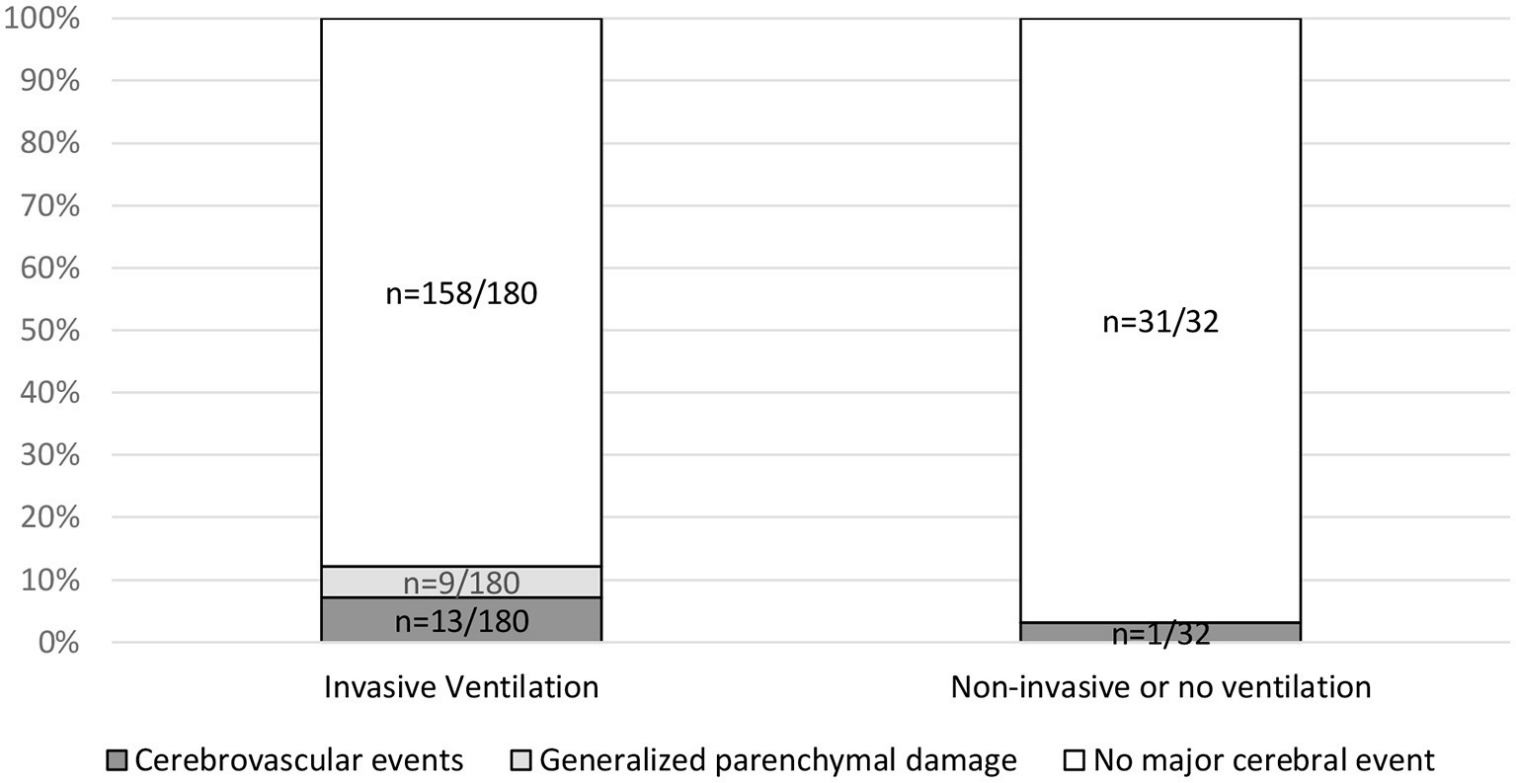
Association of major cerebral events with invasive ventilation. Invasive ventilation includes assisted pressure release ventilation (APRV) and volume controlled continuous mandatory ventilation (VC-CMV). Non-invasive ventilation includes nasal oxygen or continuous positive airway pressure (CPAP) mask. Contingency tables analysis was used to relate ventilation to the parameters cerebrovascular events (*p* = 0.39), generalized parenchymal damage (*p* = 0.20) and major events (*p* = 0.13). Information on the type of ventilation performed was available in 211 of 278 cases.

No statistically significant association between the referring ward and cerebrovascular events was found (*p* = 0.25). No statistically significant association between major cerebral events and ventilation was found (*p* = 0.13) (Figure 4). A significant association between low GCS (≤8) and invasive ventilation (81.5% as compared with 18.5% in patients with GCS >9) was confirmed with *p* < 0.001.

Coagulation parameters were tested for association with major cerebrovascular events: neither the INR (*p* = 0.60), aPTT (*p* = 0.77), nor the thrombocyte count (*p* = 0.51) were significantly different between patients with vs. those without major cerebrovascular event.

### Imaging Prediction of Patient Outcomes

**Figure 5:**
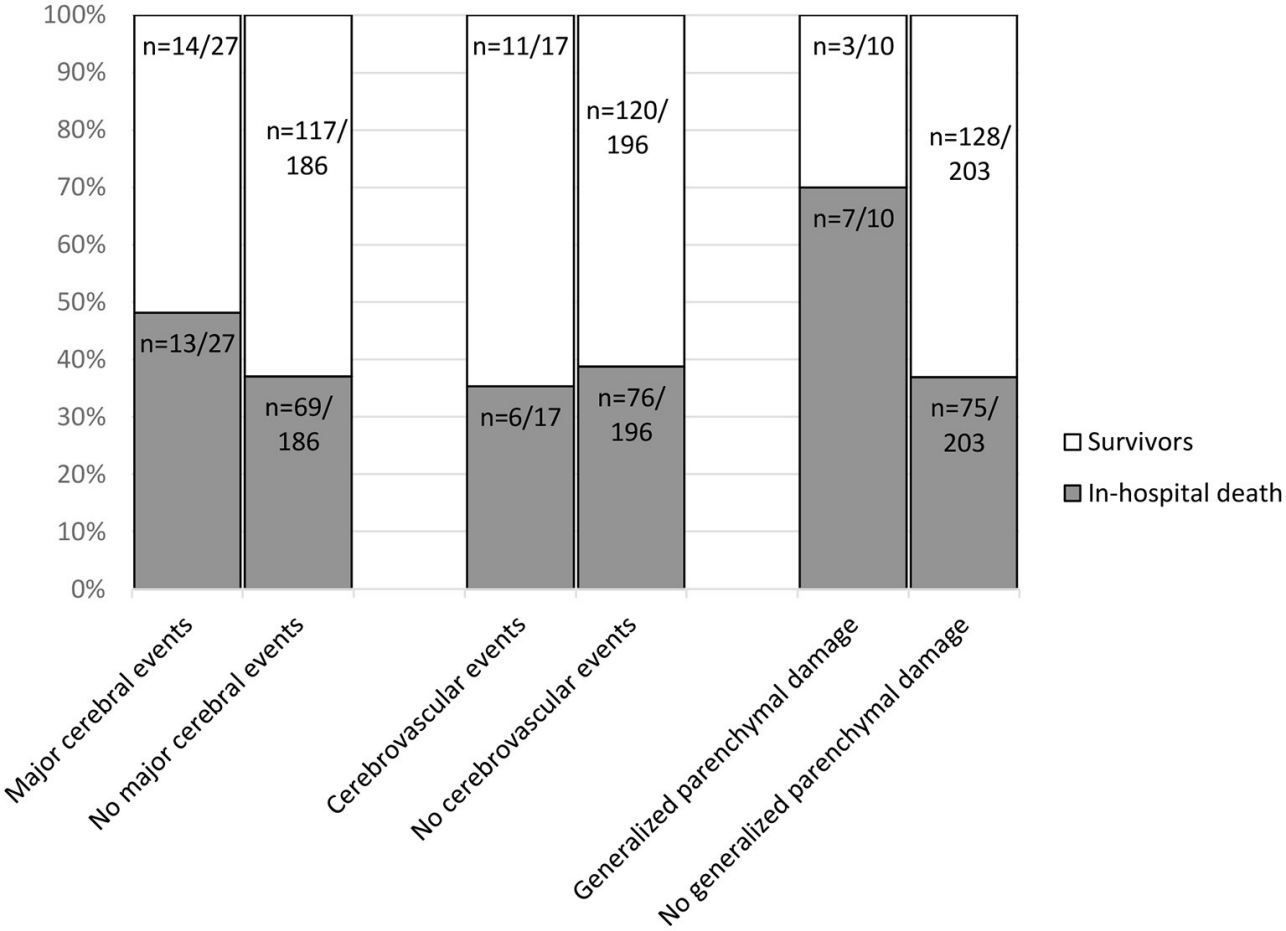
In-hospital death associated with image findings in *n* = 213 patients in a total of 278 CT examinations. The number of cerebrovascular events per patient (*n* = 17/213) differs from the previously mentioned number of cerebrovascular events in CT-scans in Table 2 (*n* = 20/278). Reason being, that some patients received multiple CT-scans. For the calculation of in-hospital death every patient was only included once. Contingency tables were used to compare the outcome parameter in-hospital death for patients with and without specific imaging findings. No statistically significant association was found with major cerebral events (*p* = 0.27). Also, cerebrovascular events were not significantly associated with mortality (*p* = 0.77). A significant association was established between in-hospital death and generalized parenchymal damage (*p* = 0.04) with an in-hospital mortality of 70 % (*n* = 7/10) in patients with generalized parenchymal damage vs. 36.9% (*n* = 75/203) in patients without generalized parenchymal damage.

In-hospital death was not significantly associated with major cerebral events (*p* = 0.27). Also, cerebrovascular events were not significantly associated with mortality (*p* = 0.77). A significant association was established between in-hospital death and generalized parenchymal damage (*p* = 0.04) with an in-hospital mortality of 70.0 % (*n* = 7/10) in patients with generalized parenchymal damage vs. 36.9% (*n* = 75/203) in patients with no generalized parenchymal damage (Figure 5).

## Discussion

### Summary

In this study, we retrospectively analyzed the occurrence of cerebrovascular events in a population of patients with sepsis who underwent full-body CT. Overall, major cerebral events were detected in 11.8% of the CT examinations. Major cerebral findings were associated with severe coma of the patient. Also, the referring ward was identified to be associated with generalized parenchymal damage but not cerebrovascular events. Generalized parenchymal damage but not cerebrovascular events was associated with in-hospital mortality.

### Literature

The role of full-body CT in septic patients has been studied extensively with respect to focus identification and its impact of patient management (14, 15). Previously, our group has shown the accuracy of focus prediction to depend on CT readers' confidence in the diagnosis of sepsis using structured analysis of CT reports (^*^ blinded for submission). Another group has reported an association between stroke and sepsis (8). Although the significance of cerebrovascular events for managing patients with sepsis seems evident, a systemic and detailed analysis of their detection on cCT performed along with full-body CT was not available before. Another diagnostic caveat would also be the clinical distinction between SAE and stroke (4, 21). As pointed out by Sonneville et al., the prevalence of prior stroke is more common in patients with SAE (7.4%) as opposed to septic patients with no SAE (3.9%) suggesting a possible association of both stroke and SAE. Still, a lack of data providing the occurrence of new cerebrovascular events in the course of sepsis can be appreciated (4). Contrary to previous reports, our study failed to show a prognostic relevance of cerebrovascular events probably due to the small sample size. As coagulopathy has been described as both prognostically relevant and an important target for medical intervention in patients with sepsis, its association with cerebrovascular events in sepsis deserves further investigation (6, 22, 23). Further data on imaging correlates of sepsis-induced neurologic dysfunction on CT and MRI and their diagnostic accuracy are required.

### Limitations

The retrospective design of this study limits interpretation of the results. The above-mentioned associations of imaging features and patient outcomes demand critical discussion and prospective analysis before being interpreted as causalities. Furthermore, cCT is known to be an insufficient imaging technique for detecting SAE – a common sequela of severe infection and intensive care. Importantly, patients in our study population did not necessarily have prior brain imaging, to define their individual baseline white matter status. As this study focused on the occurrence of cerebrovascular events in septic patients, a more accurate analysis of parenchymal alterations including MRI findings during sepsis is beyond the scope of this manuscript. In our opinion, the number of cerebrovascular events in this patient population may be too small to confirm the above mentioned prognostic role in sepsis. This study was planned and conducted to define the incidence of such events, and we additionally studied the occurrence of generalized parenchymal damage to account for another major cerebral finding typically seen in septic patients after resuscitation. One drawback of our retrospective analysis is the lack of documentation of narcotic agents to cross-reference with GCS. Still, the need for sedation reflects the neurological impairment in a broader sense. A further analysis of the infectious source identified on body CT was not subject of the present study, as this will be analyzed in a separate study. The role of acute intracranial hemorrhage in patients with severe acute respiratory syndrome coronavirus 2 (SARS-CoV-2) infection (COVID-19) has recently been described by our group (24). A more detailed analysis of cCT findings in patients with COVID-19 is currently ongoing.

## Conclusion

This study provides further evidence that patients with suspected sepsis may additionally suffer from cerebrovascular events that are relevant for their management. We identified an association of both severe coma and the referring ward (medical/surgical) with acute cerebral findings. Besides, generalized parenchymal damage was associated with poor prognosis. Full-body CT has the advantage of both detecting septic foci and possibly identifying ischemic or hemorrhagic stroke in this high-risk patient population. Prospective trials should evaluate clinical decision-making tools for selecting the best imaging modality in septic patients and confirming the prognostic relevance of specific CT findings.

## Data Availability Statement

The datasets presented in this article are not readily available because local data protection is extremely strict, therefore, patient related data cannot be made available. Requests to access the datasets should be directed to JP, julian.pohlan@charite.de.

## Ethics Statement

The studies involving human participants were reviewed and approved by Ethikkommission Charité. Written informed consent for participation was not required for this study in accordance with the national legislation and the institutional requirements.

## Author Contributions

JP developed the project and coordinated the manuscript. JN performed the rating and co-wrote the manuscript. DW discussed the clinical parameters and co-wrote the manuscript. LS prepared and analyzed the data and co-wrote the manuscript. FK, JS, and SG analyzed the data and co-wrote the manuscript. RA provided a data analysis approach and co-wrote the manuscript. KR provided statistical expertise and co-wrote the manuscript. MD provided senior advice to the project and co-wrote the manuscript. All authors contributed to the article and approved the submitted version.

## Conflict of Interest

The authors declare that the research was conducted in the absence of any commercial or financial relationships that could be construed as a potential conflict of interest.

## Publisher's Note

All claims expressed in this article are solely those of the authors and do not necessarily represent those of their affiliated organizations, or those of the publisher, the editors and the reviewers. Any product that may be evaluated in this article, or claim that may be made by its manufacturer, is not guaranteed or endorsed by the publisher.

## Supplementary Material

Supplementary Figure 1 Different cerebrovascular events identified by non- contrast cCT. Ischemic stroke in the left posterior middle cerebral artery territory **(A)**; large intracerebral hemorrhage of the right parieto-occipital lobe **(B)**; left frontal subdural hematoma **(C)**; right superficial intracerebral bleeding with associated subarachnoid bleeding due to previously unknown mass lesion **(D)**. The patient in **(B)** died during the hospitalization, whereas all other patients survived.
