# Supplementary figures and images for: Cerebrovascular Events in Suspected Sepsis: Retrospective Prevalence Study in Critically Ill Patients Undergoing Full-Body Computed Tomography
Source: Front Neurol. 2022 May 9;13:811022. doi: 10.3389/fneur.2022.811022 (PMC9125158; doi:10.3389/fneur.2022.811022)

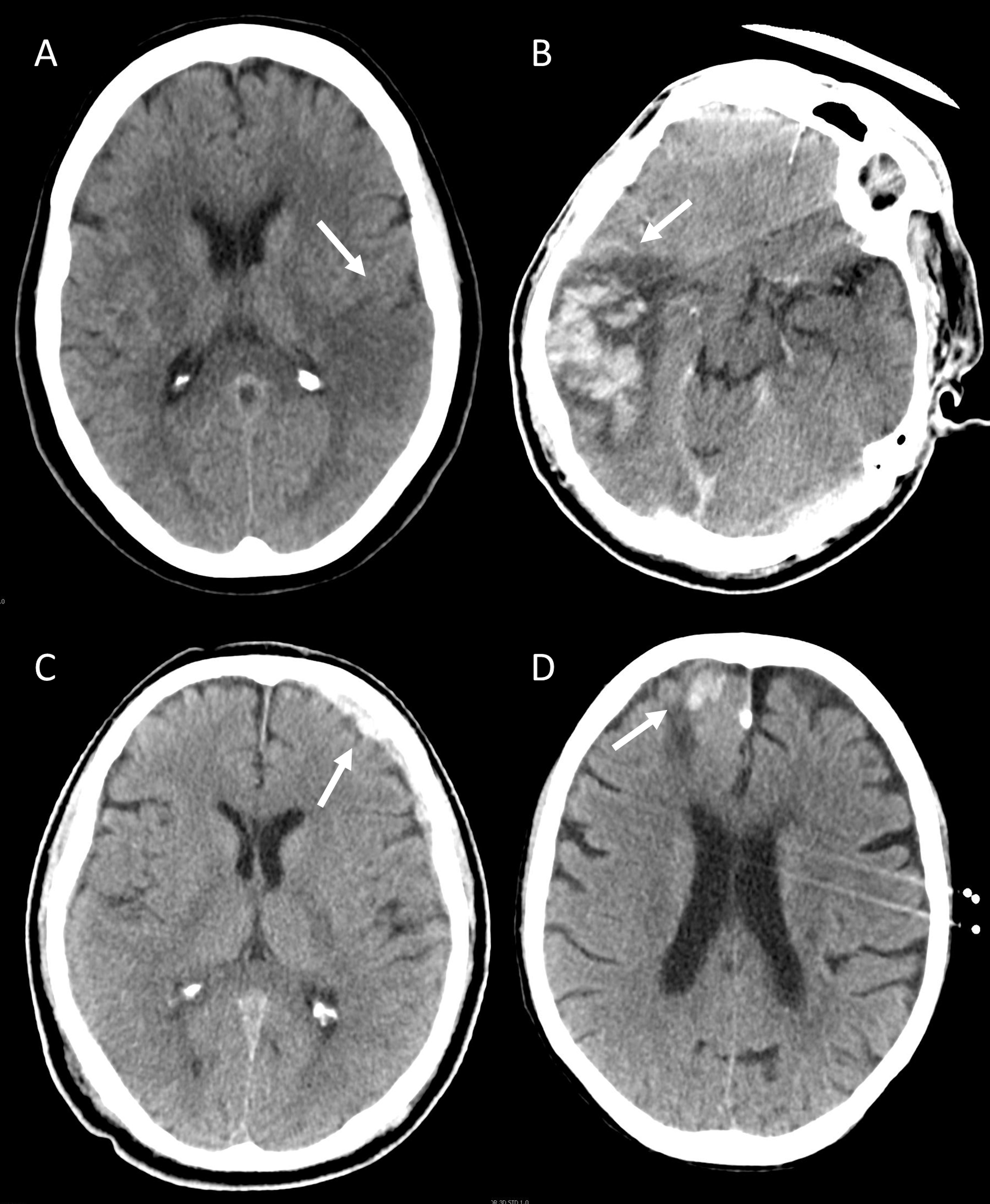

Supplement: Supplementary Figure 1 — Different cerebrovascular events identified by non- contrast cCT. Ischemic stroke in the left posterior middle cerebral artery territory (A); large intracerebral hemorrhage of the right parieto-occipital lobe (B); left frontal subdural hematoma (C); right superficial intracerebral bleeding with associated subarachnoid bleeding due to previously unknown mass lesion (D). The patient in (B) died during the hospitalization, whereas all other patients survived. [file Image_1.jpeg]
