# Supplementary material for: Cerebrovascular Events in Suspected Sepsis: Retrospective Prevalence Study in Critically Ill Patients Undergoing Full-Body Computed Tomography
Source: Front Neurol. 2022 May 9;13:811022. doi: 10.3389/fneur.2022.811022 (PMC9125158; doi:10.3389/fneur.2022.811022)
Supplement: Supplementary file 2 [file Table_1.docx]

**Supplementary table 1**: List of the categories used for review of CT reports.

| **Category** | **Description** |  |
| --- | --- | --- |
| Cerebrovascular events | Stroke or intracerebral hemorrhage | |
| Mass lesion | Lesion with midline shift or herniation | |
| Generalized parenchymal damage | Hypoxic brain damage/diffuse brain swelling | |
| Atrophy | Involution of brain parenchyma | |
| White matter disease | Leukencephalopathy or lacunas | |
| Vasosclerosis | Calcification/atherosclerosis of cerebral blood vessels | |
| Small mass lesion | Lesion without midline shift or herniation | |
| Ventricular pathology | Hydrocephalus/dilated ventricles | |
| Chronic vascular condition | Parenchymal damage caused by preceding cerebrovascular event | |
